# Integrated Inflammatory and Gut Microbial Signatures in Major Depressive Disorder: A Case–Control Study

**DOI:** 10.3390/brainsci16070681

**Published:** 2026-06-28

**Authors:** Nour Dabboussi, Espérance Debs, Marc Bouji, Raymond Kassab, Rami Bou Khalil, Nassim Fares, Rayane Rafei

**Affiliations:** 1 Laboratory of Research in Physiology and Pathophysiology, Faculty of Medicine, Saint Joseph University of Beirut, Beirut 1107 2020, Lebanon; nour.dabboussi@net.usj.edu.lb; 2 Laboratoire Microbiologie Santé et Environnement (LMSE), Doctoral School of Science & Technology, Faculty of Public Health, Lebanese University, Tripoli 1300, Lebanon; 3 Department of Biology, Faculty of Arts and Sciences, University of Balamand, Tripoli P.O. Box 100, Lebanon; esperance.debs@balamand.edu.lb; 4 Faculty of Sciences, Saint Joseph University of Beirut, Beirut 1104 2020, Lebanon; marc.bouji@usj.edu.lb; 5 Department of Psychiatry, Hôtel-Dieu de France Hospital, Saint-Joseph University of Beirut, Beirut P.O. Box 11-5076, Lebanon; ray.kassab@yahoo.com (R.K.); rami.boukhalil@usj.edu.lb (R.B.K.)

**Keywords:** major depressive disorder, gut microbiota, inflammation, C-reactive protein, interleukin-6, 16S rRNA sequencing, Lebanese population

## Abstract

**Highlights:**

**What are the main findings?**

- Depression was not independently associated with IL-6 or CRP; BMI (adiposity proxy) was the main determinant of CRP.
- MDD was linked to selective gut microbiota shifts at the genus level, with beta diversity analyses revealing modest metric-dependent differences.

**What are the implications of the main findings?**

- Adiposity, rather than depression itself, may drive systemic inflammation in MDD, suggesting the need to account for BMI in future studies.
- This study provides the first integrated analysis of gut microbiota and inflammation in Lebanese adults with MDD, highlighting the importance of population-specific microbiome research.

**Abstract:**

Background/Objectives: Major depressive disorder (MDD) is increasingly recognized as involving inflammation and the microbiota–gut–brain axis. Few studies have simultaneously assessed systemic inflammatory markers and gut microbiota composition within the same cohort while accounting for metabolic confounders. Moreover, data from Middle Eastern and North African (MENA) populations remain limited, restricting our understanding of how diet may influence neuroimmune–microbiome interactions in depression. This study aimed to investigate associations between MDD, systemic inflammatory markers, and gut microbiota composition in Lebanese adults. To our knowledge, this is the first study of its kind in Lebanon, as well as in the MENA region. Methods: In this cross-sectional case–control study, we examined circulating inflammatory markers and gut microbial profiles in 46 adults with DSM-5-confirmed MDD and 25 healthy controls. Plasma C-reactive protein (CRP) and interleukin-6 (IL-6) were measured, and the gut microbiota composition was characterized using 16S rRNA gene sequencing. Multivariable models were adjusted for age, sex, body mass index (BMI), Mediterranean diet adherence, and fluoxetine exposure. Results: Depression status was not independently associated with CRP or IL-6 after adjustment, whereas BMI emerged as a significant determinant of systemic inflammation. At the genus level, MDD was associated with the enrichment of *Dorea*, *Lachnoclostridium*, *Collinsella*, *Bilophila*, and *Klebsiella* and the depletion of *Christensenella*, *Mitsuokella*, and *Victivallis*, independent of inflammatory biomarkers. Alpha diversity did not differ between groups, while beta diversity showed modest metric-dependent differences, primarily driven by presence/absence-based measures. Conclusions: Specific microbial taxa may contribute to gut–brain signaling pathways implicated in MDD and systemic inflammation. Further longitudinal and mechanistic studies are required to clarify causal interactions within inflammation–microbiome networks in MDD.

## 1. Introduction

Major depressive disorder (MDD) is a complex multifactorial condition resulting from interactions between genetics, epigenetic regulation, and environmental stresses [1,2]. It has been increasingly conceptualized within a neuroimmune framework, in which peripheral inflammatory signaling interacts bidirectionally with the central nervous system (CNS). Elevated circulating pro-inflammatory cytokines, including interleukin-6 (IL-6), as well as acute-phase proteins such as C-reactive protein (CRP), have been consistently associated with depressive symptoms [2,3,4,5]. Mechanistically, peripheral immune activation may influence CNS function through the cytokine-mediated modulation of blood–brain barrier permeability, microglial priming, and alterations in monoaminergic neurotransmission, including activation of the tryptophan–kynurenine pathway [6,7,8,9]. However, inflammatory alterations in MDD have not been consistently observed across studies. The effect sizes are generally modest, and a substantial proportion of patients do not exhibit elevated inflammatory markers. One factor that may contribute to this heterogeneity is the metabolic status. Adiposity, in particular, is a well-established driver of low-grade systemic inflammation and is independently associated with CRP and IL-6 levels [4,10]. Consequently, inadequate adjustment for metabolic variables may confound associations between inflammation and depression, complicating the interpretation of whether inflammatory activation is intrinsic to MDD or secondary to metabolic dysregulation.

Beyond systemic inflammatory mechanisms, growing attention has been paid to the gut microbiota as a key regulator of neuroimmune communication within the gut–brain axis [11,12]. Through short-chain fatty acid (SCFA) production, the regulation of intestinal barrier integrity, lipopolysaccharide (LPS)-mediated immune translocation, vagal pathways, and immune system calibration, the gut microbiota may influence the function and behavior of the CNS [10,13,14,15]. Studies in MDD have reported the enrichment of potentially pro-inflammatory taxa alongside the depletion of SCFA-producing commensals; however, specific microbial signatures vary substantially across cohorts [16,17,18,19]. Meta-analytic evidence supports alterations in the microbial composition in MDD but highlights considerable inter-study heterogeneity, likely reflecting differences in diet, metabolic status, geographic background, and analytical approaches [19].

Twin studies estimate the heritability of MDD at approximately 30–40%, indicating that, although genetic factors contribute substantially to disease susceptibility, environmental exposures play a critical role in shaping individual risk [20]. Genome-wide association studies (GWASs) have identified a polygenic architecture involving numerous variants of small effect related to serotonergic neurotransmission, synaptic plasticity, and stress response pathways. In parallel, environmental stressors—particularly early-life adversity—can induce epigenetic modifications, including DNA methylation, histone modifications, and non-coding RNA alterations, which may dysregulate the hypothalamic–pituitary–adrenal (HPA) axis and related neurobiological systems [1,21]. Stress-related epigenetic alterations involving glucocorticoid signaling genes such as *NR3C1* and *FKBP5* have been associated with altered stress responsivity and increased vulnerability to depression. Together, these gene–environment interactions provide a biological framework through which cumulative psychosocial stress may confer long-term susceptibility to depressive disorders [21].

These mechanisms may be particularly relevant in populations exposed to chronic societal and traumatic stressors. The Lebanese context is therefore of particular interest, as Lebanon has experienced decades of political instability, armed conflict, economic hardship, the 2020 Beirut Port explosion, and the COVID-19 pandemic [22]. In a nationally representative study conducted in 2022 among 1000 Lebanese adults, 47.8% screened positive for probable depression [23]. This persistent population-level adversity may contribute to HPA axis sensitization, epigenetic stress embedding, and downstream neuroimmune dysregulation, potentially contributing to the high burden of depressive symptoms observed in this population [21].

Despite this context, the biological correlates of MDD within the Lebanese population remain largely unexplored. Relatively few studies have simultaneously assessed systemic inflammatory markers and gut microbiota composition within the same cohort while rigorously adjusting for metabolic confounders [24,25]. Integrating these domains is essential to determine whether microbial alterations associated with MDD persist independently of systemic inflammatory activation and metabolic factors. Furthermore, data from Middle Eastern and North African populations in general remain limited, restricting the generalizability of the current findings and limiting our understanding of how dietary factors may shape neuroimmune–microbiome interactions in depression.

Accordingly, the aim of the present study was to examine circulating inflammatory markers (CRP and IL-6) and gut microbial composition in Lebanese adults with MDD compared with healthy controls, and to determine whether microbial signatures are associated with depression status after accounting for systemic inflammatory markers.

## 2. Materials and Methods

### 2.1. Study Design and Ethical Approval

This observational cross-sectional study with a case–control design was registered at ClinicalTrials.gov (NCT05646784) and conducted at the Hôtel-Dieu de France Hospital and affiliated clinics in North Lebanon between January 2024 and January 2025. The study protocol was approved by the institutional ethics committee of the Hôtel-Dieu de France (CEHDF 2009). All participants provided written informed consent prior to enrollment, and the study was conducted in accordance with the Declaration of Helsinki and local regulations governing clinical research.

### 2.2. Participants

Men and women aged 18–65 years were recruited into either an MDD group or a healthy control group.

Participants in the MDD group (*n* = 46) were experiencing a current major depressive episode, confirmed using the Mini International Neuropsychiatric Interview (MINI) according to the Diagnostic and Statistical Manual of Mental Disorders, fifth edition (DSM-5) criteria [26]. The depression severity was assessed using the Montgomery–Åsberg Depression Rating Scale (MADRS), and a score of ≥20 was required for inclusion [27]. Healthy controls (*n* = 25) were recruited via online pre-screening; the absence of current or past MDD was confirmed using the MINI and MADRS.

The exclusion criteria for both groups included antibiotic use within 4 weeks prior to sampling; current treatment with anti-inflammatory or immunosuppressive agents; laxative use; pregnancy or breastfeeding; and comorbid psychiatric disorders (substance use disorder, bipolar disorder, or schizophrenia). To balance the external validity and ensure our sample reflected the natural heterogeneity of MDD in clinical settings, we placed no restrictions on the number of previous depressive episodes or historical treatment resistance. All participants were required to provide stool and blood samples and complete clinical assessments.

### 2.3. Clinical and Biological Assessment

Demographic and clinical data were collected, including age, sex, BMI, lifestyle factors, medication use, and dietary adherence, which was assessed using the 14-item Mediterranean Diet Adherence Screener [28]. All participants underwent psychiatric evaluation (MINI and MADRS), blood sampling, and stool collection. Venous blood (10 mL) was collected into EDTA tubes and processed within 2 h, and plasma was aliquoted and stored at −80 °C until analysis. Plasma IL-6 concentrations were measured using the Elecsys IL-6 electrochemiluminescence immunoassay (ECLIA) on the cobas e801 analyzer (Roche Diagnostics GmbH, Mannheim, Germany) (analytical range: 1.5–5000 pg/mL; sensitivity < 1.5 pg/mL). CRP concentrations were measured using the MULTIGENT CRP Vario latex-enhanced immunoturbidimetric assay (Abbott Diagnostics, Abbott Park, IL, USA) on an ARCHITECT c16000 analyzer. The analytical measuring range was 0.2–320 mg/L.

### 2.4. Stool Processing, Sequencing, and Bioinformatic Analyses

#### 2.4.1. Sample Collection and DNA Extraction

Participants collected fresh stool samples in sterile containers and delivered them to the laboratory within 2 h. Upon receipt, the samples were stored at 4 °C, homogenized, aliquoted, and stored at −80 °C until processing. Genomic DNA was extracted using the QIAamp DNA Stool Mini Kit (QIAGEN GmbH, Hilden, Germany), according to the manufacturer’s instructions. DNA purity was assessed using a NanoDrop spectrophotometer (Thermo Fisher Scientific, Waltham, MA, USA), and the concentrations were re-quantified fluorometrically using a Qubit fluorometer (Thermo Fisher Scientific, Waltham, MA, USA) prior to library preparation.

#### 2.4.2. 16S rRNA Gene Amplification and Sequencing

The V3–V4 region of the bacterial 16S rRNA gene was amplified using universal primers [29]. The PCR reactions (25 µL) contained 50 ng genomic DNA, 1× KAPA HiFi HotStart ReadyMix (Roche Sequencing Solutions, Pleasanton, CA, USA), and 200 nM of each primer. The thermal cycling conditions were 95 °C for 3 min; 25 cycles of 95 °C for 30 s, 55 °C for 30 s, and 72 °C for 30 s; followed by 72 °C for 5 min.

Amplicons were indexed using Nextera XT v2 index primers (Illumina, San Diego, CA, USA), pooled, normalized, and sequenced on an Illumina MiSeq platform (Illumina, San Diego, CA, USA) using the MiSeq Reagent Kit v3 (2 × 300 bp; Illumina, San Diego, CA, USA). A mock microbial community (ATCC MSA-1002; American Type Culture Collection, Manassas, VA, USA) served as a positive control, and nuclease-free water served as a negative control. All libraries were sequenced in a single MiSeq run. Samples were processed on two plates, each including the same mock community to monitor the technical variability.

#### 2.4.3. Sequence Processing and Diversity Analyses

FASTQ files were processed using QIIME 2 (2022.8). Demultiplexed paired-end reads were quality filtered, trimmed (280 bp forward, 260 bp reverse), and denoised using the DADA2 plugin (q2-dada2, version 2022.8) to generate amplicon sequence variants (ASVs) with a total of 5687 features. Taxonomy was assigned using the QIIME 2 feature-classifier plugin with the GSR reference database. Genus- and phylum-level abundance tables were generated for downstream analyses. Alpha diversity metrics (Shannon index, Pielou’s evenness, Observed Features, Faith’s Phylogenetic Diversity) and beta diversity metrics (Bray–Curtis, Jaccard, weighted and unweighted UniFrac distances) were calculated using the QIIME 2 core-metrics pipeline. Rarefaction was applied exclusively for diversity analyses at a depth of 14,529 reads. Two samples with low ASV counts were excluded. Principal coordinates analysis (PCoA) was used for ordination. Beta diversity differences were assessed using PERMANOVA with 999 permutations, and the homogeneity of dispersion was evaluated using betadisper.

### 2.5. Statistical Analyses

CRP and IL-6 were log-transformed to address right-skewed distributions. Linear regression models estimated the crude and adjusted associations between depressive status and biomarker levels. Associations between depressive status and inflammatory markers were assessed using a staged regression approach. The unadjusted models included depressive status as the sole predictor of each outcome. The adjusted models additionally included age, sex, BMI, Mediterranean diet adherence, and binary fluoxetine exposure (yes/no) as covariates, selected a priori, based on their known associations with systemic inflammation and depression. This approach allowed for the assessment of both crude associations and associations independent of potential confounders. The continuous fluoxetine (mg/day) dose was additionally included as a covariate in analyses restricted to participants with depression. All covariates were entered simultaneously into the regression models.

For microbiome analyses, relative abundances were log10-transformed after addition of a pseudocount prior to regression modeling. Logistic regression models evaluated the associations between microbial features (alpha diversity, beta diversity, and relative abundance) and depressive status, adjusting for age, sex, BMI, and Mediterranean diet adherence, which were selected a priori based on their known associations with the gut microbiome and depression. Variance inflation factors were calculated to assess multicollinearity. Beta diversity associations were examined using PERMANOVA models including depressive status and covariates, with effect sizes reported as R^2^ values. All analyses were conducted using Stata version 17 (StataCorp, College Station, TX, USA), SPSS version 29 (IBM, Armonk, NY, USA), and R version 4.3.1 (R Foundation for Statistical Computing, Vienna, Austria) with the vegan and ggplot2 packages. Statistical significance was defined as two-sided α = 0.05.

### 2.6. Outcomes

The primary outcomes were plasma CRP (mg/L) and IL-6 (pg/mL). Secondary outcomes included gut microbiome alpha diversity indices, beta diversity metrics, and genus-level relative abundances.

### 2.7. Sample Size

The sample size was calculated a priori using G*Power 3.1 [30] for linear multiple regression (fixed model, R^2^ increase) based on the primary outcomes (CRP and IL-6). Assuming a medium effect size (f^2^ = 0.15), α = 0.05, power = 0.80, one tested predictor, and four total predictors, 55 participants were required. To account for potential dropouts and protocol violations, 73 participants were enrolled (48 MDD, 25 controls). The effect size estimates were based on previous studies reporting associations between inflammation markers and depressive status (CRP: mean ≈ 2.5 mg/L, SD ≈ 1.2; IL-6: mean ≈ 3.1 pg/mL, SD ≈ 1.5) [31,32]. After excluding two participants due to protocol violations, 71 participants (46 MDD, 25 controls) were included in the final analysis. Calculations were performed separately for CRP and IL-6, and the larger required sample size was retained to ensure adequate power for both outcomes.

## 3. Results

### 3.1. Participant Characteristics

Women predominated in both groups (78.3% in MDD vs. 80.0% in controls; *p* = 0.92, χ^2^ test). The mean age was higher in the MDD group (36.52 ± 10.84 years) than in the controls (27.88 ± 8.35 years; *p* < 0.001, independent *t*-test). The body mass index (BMI) was comparable between groups (24.56 ± 4.91 kg/m^2^ vs. 25.14 ± 4.65 kg/m^2^; *p* = 0.58, independent *t*-test). Participants with MDD reported substantially lower levels of physical activity, with 93.5% reporting minimal or no exercise compared with 48.0% of controls (*p* < 0.001, χ^2^ test). Adherence to the Mediterranean diet was similar between groups (*p* = 0.34, independent *t*-test).

### 3.2. Inflammatory Biomarkers

The mean plasma CRP concentrations were higher in participants with MDD, and the IL-6 levels were slightly higher compared with the controls, but neither difference was statistically significant. Detailed descriptive statistics are presented in Table 1.

#### 3.2.1. Plasma IL-6 Is Not Independently Associated with Depression

In the multivariate linear regression models (Table 2), depressive status was not associated with the IL-6 levels (adjusted β = 0.08, 95% CI −0.24 to 0.40, *p* = 0.62). In the unadjusted analyses, the BMI (β = 0.03, *p* = 0.041) and age (β = 0.02, *p* = 0.002) were positively associated with IL-6 concentrations. However, these associations were attenuated after adjustment. In the fully adjusted model, age showed a borderline positive association (adjusted β = 0.01 per year, 95% CI −0.00 to 0.03, *p* = 0.10), while the BMI was no longer significant (adjusted β = 0.02, 95% CI −0.02 to 0.06, *p* = 0.26). Sex and Mediterranean diet adherence were not associated with IL-6 levels.

#### 3.2.2. Plasma CRP Levels Are Influenced by BMI but Not Depression Status

The depressive status was not associated with CRP concentrations in the adjusted analyses (adjusted β = 0.15, 95% CI −0.50 to 0.80, *p* = 0.65). However, the BMI remained independently associated with CRP. Each 1 kg/m^2^ increase in BMI was associated with higher CRP levels (adjusted β = 0.08, 95% CI 0.00 to 0.15, *p* = 0.038). Age and sex were not significantly associated with CRP. Mediterranean diet adherence showed a non-significant inverse association (adjusted β = −0.18, *p* = 0.22) (Table 3).

#### 3.2.3. Multivariate Modeling of IL-6 and CRP: Depression Status Is Not a Significant Predictor

Multivariate linear regression, jointly modeling IL-6 and CRP, yielded results consistent with the separate models. Depressive status was not independently associated with either biomarker. The BMI remained positively associated with CRP but not IL-6. Age demonstrated a positive but non-significant association with IL-6 and no association with CRP. Mediterranean diet adherence was not independently associated with either inflammatory marker (Table 4).

### 3.3. Microbiome Composition

#### Genus-Level Gut Microbiota Associated with Depressive Status

After adjustment for age, sex, BMI, Mediterranean diet adherence, and inflammatory biomarkers, ten genera remained independently associated with the depressive status (Supplementary Table S1). Depression was associated with a higher relative abundance of *Dorea*, *Bilophila*, *Collinsella*, *Lachnoclostridium*, *Klebsiella*, and *Negativibacillus* and a lower abundance of *Mitsuokella*, *Christensenella,* and *Victivallis*. Other taxa that were significant in the unadjusted analyses did not maintain their associations after multivariate adjustment (see Supplementary Table S1).

At the phylum level, depression was independently associated with a lower abundance of *Lentisphaerae* and *Tenericutes* (Supplementary Table S2).

### 3.4. Microbial Diversity

#### 3.4.1. Alpha Diversity

No significant differences were observed between groups for Shannon entropy (*p* = 0.789), Pielou’s evenness (*p* = 0.384), Observed Features (*p* = 0.679), or Faith’s Phylogenetic Diversity (*p* = 0.108). In the multivariable linear regression models, depressive status was not associated with Faith’s index (β = −1.323, *p* = 0.343), nor were covariates independently associated with phylogenetic diversity (Table 5).

#### 3.4.2. Beta Diversity

PERMANOVA models demonstrated significant overall associations for the Bray–Curtis (R^2^ = 11.4%, *p* = 0.021), unweighted UniFrac (R^2^ = 11.4%, *p* = 0.015), and Jaccard distance (R^2^ = 19.3%, *p* = 0.001). Weighted UniFrac was not significant (*p* = 0.27). Marginal analyses indicated a borderline association between depressive status and the unweighted UniFrac distance (*p* = 0.052) and a significant association in the Jaccard model (*p* = 0.001). However, the Jaccard model demonstrated heterogeneous dispersion (*p* < 0.01), warranting cautious interpretation.

Sex was significantly associated with the unweighted UniFrac distance (*p* = 0.048). Age, BMI, Mediterranean diet adherence, and fluoxetine dose were not independently associated with beta diversity metrics (Supplementary Tables S3–S6).

**Figure 1:**
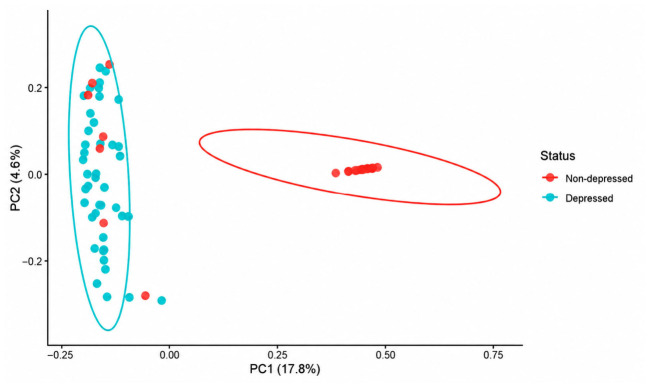
PCoA of gut microbiota beta diversity based on the Jaccard distance in depressed patients and healthy controls. Statistical differences were assessed using marginal PERMANOVA adjusted for confounders.

**Figure 2:**
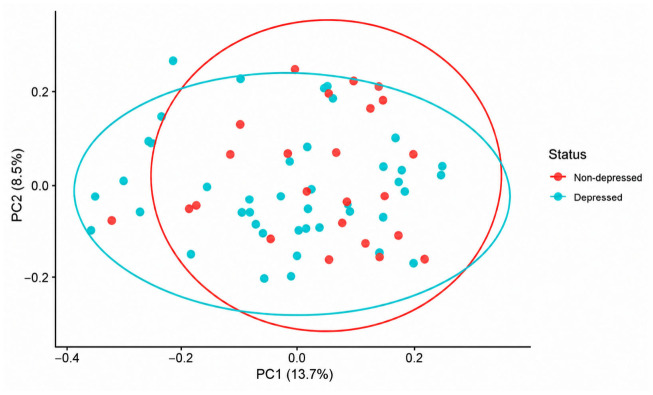
PCoA of gut microbiota beta diversity based on the unweighted UniFrac distance in depressed patients and healthy controls. Statistical differences were assessed using marginal PERMANOVA adjusted for confounders.

PCoA showed substantial overlap between groups, with partial separation observed primarily in presence/absence-based metrics (Jaccard and unweighted UniFrac) (Figure 1 and Figure 2 and Supplementary Figures S1–S3).

## 4. Discussion

In this cross-sectional case–control study, we investigated the relationships between MDD, systemic inflammatory markers (IL-6 and CRP), and gut microbiota composition in Lebanese adults, accounting for BMI, age, sex, and diet confounders. There were three principal findings. First, depression status was not independently associated with circulating IL-6 or CRP after adjustment, whereas BMI emerged as a consistent determinant of systemic inflammation. Second, the gut microbiota analysis revealed selective genus-level compositional differences between depressed patients and healthy controls, without alterations in overall alpha diversity. Third, beta diversity analyses indicated modest but significant differences driven primarily by presence–absence metrics, suggesting subtle shifts in microbial community structure.

Together, these findings suggest that, in this population, depression is characterized more by specific microbial alterations than by overt systemic inflammation, which highlights the importance of the metabolic context—particularly adiposity—in shaping inflammatory profiles. This integrative approach provides novel insight into microbiota–immune interactions in MDD within an underrepresented Middle Eastern population.

Our results align with meta-analytic evidence suggesting that associations between depression and CRP or IL-6 are small and often attenuated after controlling for metabolic and lifestyle confounders [33,34]. Although some studies report persistent associations after BMI adjustment [4], and longitudinal data support bidirectional links between inflammatory markers and depressive symptoms [35], large-scale cohorts indicate that metabolic factors substantially account for the observed inflammation differences [36]. Similarly, meta-analyses demonstrate elevated inflammatory markers in MDD compared with controls, though the effect sizes are generally modest and sensitive to adjustment [3,5]. Together, the literature suggests that inflammation in depression is heterogeneous and strongly intertwined with metabolic health.

Importantly, the absence of independent CRP or IL-6 associations in our cohort does not preclude immune involvement in MDD. Rather, these findings may align with the view that systemic inflammation is not a universal feature of depression but may characterize a distinct inflammatory subtype. According to the neuro-immune–metabolic–oxidative stress (NImetox) framework [37], depression may reflect dysregulation across interconnected immune and metabolic pathways rather than isolated elevations in nonspecific systemic markers. In this context, BMI may function not only as a confounder but also as a potential mechanistic mediator linking metabolic dysregulation to inflammatory tone.

Consistent with established biology, the BMI was significantly associated with CRP. Adipose tissue, particularly visceral fat, contributes to chronic low-grade inflammation through adipokine secretion and immune cell activation [38]. Weight-related or adiposity-related inflammation may also be amplified by metabolic endotoxemia, whereby gut-derived LPS translocation promotes systemic inflammatory signaling [39,40]. Thus, the metabolic status appears central to interpreting inflammatory phenotypes in depression [41]. Our results may also suggest that, in patients with comorbid obesity and depression, inflammation is more likely driven by adiposity rather than by the psychiatric state.

At the microbial level, we identified several genera associated with depression status. Rather than reflecting global community disruption, these changes cluster into functionally coherent patterns—enrichment of LPS-producing pathobionts, shifts in metabolic intermediary taxa, and depletion of SCFA-producing protective commensals—collectively suggesting a microbial landscape favoring pro-inflammatory signaling and reduced neuroprotective metabolite availability.

The first functional cluster comprises LPS-producing and barrier-disrupting pathobionts. *Klebsiella* enrichment is consistent with previous MDD studies and has been linked to endotoxin production and innate immune signaling [42,43,44,45,46]. Maes et al. found significantly elevated serum IgM and IgA antibodies against *Klebsiella pneumoniae* LPS in MDD patients compared to controls (ROC area 90.1%), suggesting increased bacterial translocation [44]. Likewise, *Bilophila* has been reported as elevated in MDD cohorts and is associated with barrier perturbation and host–microbe immune interactions [47,48,49]. Importantly, in the absence of independent systemic CRP or IL-6 associations in our cohort, these taxa may influence depressive phenotypes through localized gut immune signaling, epithelial integrity, and microbial metabolite production rather than overt systemic inflammatory activation. Specifically, the enrichment of pathobionts such as *Bilophila* can drive localized mucosal inflammation triggering direct neuroimmune signaling pools without elevating peripheral inflammatory markers.

A second functional cluster encompasses taxa involved in metabolic intermediary pathways, including bile acid transformation, SCFA precursor metabolism, and neurotransmitter precursor availability. The role of *Collinsella* appears context-dependent. While some studies suggest involvement in bile acid transformation and short-chain fatty acid (SCFA) metabolism [50], others associate it with inflammatory conditions such as rheumatoid arthritis [51]; altered abundance in MDD has also been reported [52]. Similarly, *Lachnoclostridium* and *Dorea* participate in microbial metabolic networks that may influence SCFA availability, neurotransmitter precursor pathways, and gut barrier dynamics [50,53,54]. These functions are increasingly recognized as relevant to gut–brain communication beyond systemic inflammatory markers.

The third and functionally most significant cluster comprises depleted SCFA-producing and gut barrier-supportive commensals. Depletion of *Christensenella*, a genus associated with SCFA production and gut barrier support, aligns with the literature suggesting reduced protective microbial functions in MDD [55]. Reduced *Mitsuokella* and *Victivallis* may further indicate diminished SCFA-mediated and mucosal-supportive pathways [56,57]. The reduction in SCFA-producing bacteria suggests a loss of butyrate-mediated neuroprotection. SCFAs play a key role in maintaining blood–brain barrier integrity and regulating inflammation in the central nervous system [58,59]. Collectively, these genus-level findings suggest alterations in microbial taxa involved in barrier integrity, metabolic signaling, and host–microbe interactions rather than evidence of widespread ecological collapse or uniform systemic inflammatory activation. In Lebanese adults, where the baseline gut microbiota composition remains poorly characterized at the population level, and dietary, environmental, and lifestyle factors differ from many Western cohorts, these selective microbial shifts may reflect region-specific configurations of gut–brain interactions in MDD.

Alpha diversity did not differ between groups across multiple indices, and adjusted analyses confirmed the absence of a significant association. This accords with systematic reviews noting inconsistent or null associations between alpha diversity and MDD [17,60], suggesting that overall richness may be less informative than compositional shifts.

Beta diversity analyses revealed modest metric-dependent differences. Presence/absence-based metrics (unweighted UniFrac, Jaccard) demonstrated significant global effects, whereas abundance-weighted measures showed weaker or non-significant associations, paralleling heterogeneous findings in the literature [2,31,61]. These patterns suggest that depression-related signals may primarily involve qualitative shifts in low-abundance taxa rather than large-scale abundance changes. The substantial overlap observed in the PCoA space further indicates that the group differences are subtle. Sex also contributed to microbiota variation, consistent with the evidence that hormonal and behavioral factors influence microbial composition [62].

Despite the high prevalence of depressive symptoms reported in the Lebanese population, published research directly examining gut microbiota composition in MDD within Lebanon remains absent, highlighting a significant gap that the present study addresses. Although a few small studies have profiled the gut microbiota in lean versus obese Lebanese adults [63] or included limited healthy control groups in disease-focused investigations [64], the large-scale characterization of gut microbial composition in healthy Lebanese adults is lacking. This underscores the novelty of the present study in providing a normative context for microbiota–depression associations in this population. Furthermore, the specific psychosocial landscape of Lebanon introduces distinct biological pressures. Prolonged exposure to macro-level stressors may drive sustained activation of the HPA axis, elevating systemic cortisol levels that can directly compromise intestinal epithelial tight junctions, alter the local mucosal immune environment, and selectively favor the survival of pathobionts over protective commensals. These unique environmental and neuroendocrine dynamics highlight how region-specific configurations of gut–brain interactions may shape the distinct microbial phenotypes observed in our depressed cohort.

### Limitations

Several limitations warrant consideration. The cross-sectional case–control design precludes causal inference, and unbalanced group sizes may limit the power for differential abundance analyses. In addition, the absence of large population-level reference datasets for the healthy Lebanese gut microbiome limits the ability to fully contextualize our findings. Given that we performed the power analysis based on our primary outcome (inflammation), the microbiome findings might be exploratory and underpowered. Larger longitudinal cohorts integrating functional profiling approaches will be necessary to clarify the mechanistic pathways linking metabolic status, inflammation, and gut microbial ecology in depression and to determine which features are population-specific versus generalizable.

## 5. Conclusions

This study provides the first integrated assessment of systemic inflammation and gut microbiota composition in Lebanese (and MENA region) adults with MDD. In this population, adiposity (approximated by BMI) appears to contribute to individual variation in inflammatory profiles, while depression-associated microbiome alterations are evident as selective genus-level shifts affecting taxa involved in gut barrier integrity, microbial metabolism, and localized immune interactions.

This study highlights an important shift in thinking about depression. Inflammation in MDD cannot be reduced to elevated CRP levels alone. After controlling for BMI and diet, the most meaningful differences appear at the level of the gut microbiota, particularly involving taxa such as *Klebsiella* and *Bilophila*. Clinically, this reinforces the idea that metabolic health and gut health are distinct but interconnected components in the management of depression.

These findings highlight the importance of integrating adiposity-related, immune, and microbial dimensions when characterizing the inflammatory phenotype of depression within Lebanese adults and, more broadly, in Middle Eastern populations, where baseline microbiota composition and environmental exposures may differ from Western cohorts.

Future longitudinal and mechanistic studies integrating shotgun metagenomics, metabolomics, and immune profiling are needed to clarify functional pathways linking the gut microbiota, adiposity, and neuroimmune processes in MDD. Larger cohorts will be essential to validate the microbial signatures identified in this study and determine their reproducibility across populations, including region-specific features in Lebanese and other Middle Eastern adults.

## Figures and Tables

**Table 1:** Participant characteristics.

Variable	All Participants (*n* = 71)	Depressed (*n* = 46)	Healthy Controls (*n* = 25)	*p*-Value
Age (years)	33.48 ± 10.80	36.52 ± 10.84	27.88 ± 8.35	<0.001 *
Gender				0.92
Male	15 (21.1%)	10 (21.7%)	5 (20.0%)	
Female	56 (78.9%)	36 (78.3%)	20 (80.0%)	
BMI (kg/m^2^)	24.77 ± 4.79	24.56 ± 4.91	25.14 ± 4.65	0.58
Mediterranean diet				0.34
Category 1	37 (55.2%)	25 (59.5%)	12 (48.0%)	
Category 2	21 (31.3%)	12 (28.6%)	9 (36.0%)	
Category 3	2 (3.0%)	0 (0.0%)	2 (8.0%)	
Category 4	5 (7.5%)	3 (7.1%)	2 (8.0%)	
Category 5	2 (3.0%)	2 (4.8%)	0 (0.0%)	
Fluoxetine use				<0.001 *
Yes	18 (25.4%)	18 (39.1%)	0 (0.0%)	
No	53 (74.6%)	28 (60.9%)	25 (100.0%)	
Depression score	32.54 ± 7.15	32.54 ± 7.15	--	--
IL-6 (pg/mL)	5.54 ± 4.00	5.66 ± 3.93	5.32 ± 4.20	0.72
CRP (mg/L)	3.55 ± 4.58	3.82 ± 5.09	3.05 ± 3.49	0.48

* *p* < 0.05 indicates a significant difference between groups. Age, BMI, depression score, IL-6, and CRP are presented as the mean ± SD. Categorical variables are presented as *n* (%).

**Table 2:** Univariate linear regression analysis of log-transformed plasma IL-6 levels.

Predictor	Unadjusted β (95% CI)	*p*	Adjusted β (95% CI)	*p*
Treatment group (intervention vs. control)	0.17 (−0.13, 0.47)	0.255	0.08 (−0.24, 0.40)	0.623
Body mass index (kg/m^2^)	0.031 (0.0013, 0.0608)	0.041	0.021 (−0.0156, 0.0570)	0.258
Age (years)	0.020 (0.0077, 0.0327)	0.002	0.013 (−0.0025, 0.0294)	0.097
Sex (male vs. female)	−0.26 (−0.60, 0.09)	0.145	−0.08 (−0.46, 0.29)	0.661
Mediterranean diet score	−0.065 (−0.21, 0.08)	0.362	−0.068 (−0.22, 0.08)	0.362

**Table 3:** Univariate linear regression analysis of log-transformed plasma CRP levels.

Predictor	Unadjusted β (95% CI)	*p*	Adjusted β (95% CI)	*p*
Treatment group (intervention vs. control)	0.144 (−0.44, 0.73)	0.623	0.149 (−0.50, 0.80)	0.65
Body mass index (kg/m^2^)	0.076 (0.018, 0.133)	0.01	0.078 (0.0044, 0.151)	0.038
Age (years)	0.020 (−0.0052, 0.0457)	0.117	0.0016 (−0.0306, 0.0338)	0.923
Sex (male vs. female)	−0.56 (−1.22, 0.11)	0.101	−0.25 (−1.01, 0.51)	0.514
Mediterranean diet score	−0.103 (−0.39, 0.18)	0.471	−0.183 (−0.48, 0.11)	0.223

**Table 4:** Multivariate linear regression analyses of log-transformed IL-6 and CRP.

Predictor	IL-6 β (95% CI)	*p*	CRP β (95% CI)	*p*
Treatment group	0.134 (−0.187, 0.454)	0.408	0.170 (−0.464, 0.803)	0.595
Body mass index (kg/m^2^)	0.032 (−0.0018, 0.0658)	0.063	0.082 (0.0151, 0.149)	0.017
Age (years)	0.0105 (−0.0053, 0.0262)	0.189	0.0004 (−0.0307, 0.0315)	0.979
Sex (male vs. female)	−0.034 (−0.410, 0.343)	0.859	−0.231 (−0.976, 0.514)	0.538
Mediterranean diet score	−0.071 (−0.220, 0.078)	0.345	−0.184 (−0.479, 0.111)	0.217
Intercept	0.488 (−0.469, 1.446)	0.312	−0.988 (−2.882, 0.906)	0.301

**Table 5:** Comparison of gut microbiota alpha diversity (Shannon index) between depressed patients and healthy controls.

Alpha Diversity					
Alpha Diversity Indices		All Participants (*n* = 71)	Depressed (*n* = 45)	Healthy Controls (*n* = 24)	*p* Value
Pielou_evenness	Mean ± SD	0.773 ± 0.050	0.777 ± 0.049	0.766 ± 0.053	0.384
Faith_pd	Mean ± SD	17.408 ± 4.796	16.729 ± 4.255	18.682 ± 5.549	0.108
Observed features	Mean ± SD	237.087 ± 66.969	234.622 ± 68.387	241.708 ± 65.409	0.679
Shannon entropy	Mean ± SD	6.069 ± 0.648	6.084 ± 0.651	6.040 ± 0.655	0.789

## Data Availability

All data are available in the manuscript.

## Supplementary Materials

The following supporting information can be downloaded at: https://www.mdpi.com/article/10.3390/brainsci16070681/s1, Figure S1: Principal Coordinates Analysis (PCoA) of gut microbiota composition (Bray–Curtis distance). Statistical differences were assessed using marginal PERMANOVA adjusted for confounders; Figure S2: Principal Coordinates Analysis (PCoA) of gut microbiota composition (Weighted UniFrac distance). Statistical differences were assessed using marginal PERMANOVA adjusted for confounders; Figure S3: Principal Coordinates Analysis (PCoA) of gut microbiota composition (Unweighted UniFrac distance). Statistical differences were assessed using marginal PERMANOVA adjusted for confounders; Table S1: Differential abundance of bacterial genera in depressed patients versus healthy controls/*p*-values in bold indicate statistically significant differences between depressed participants and healthy controls, based on a threshold of *p* < 0.05; Table S2: Differential abundance of bacterial phyla in depressed patients versus healthy controls/ P-values in bold indicate statistically significant differences between depressed participants and healthy controls, based on a threshold of *p* < 0.05; Table S3: Marginal effects of depressive status and covariates on gut microbiota beta diversity (Bray–Curtis dissimilarity); Table S4: Marginal effects of depressive status and covariates on gut microbiota beta diversity (Jaccard dissimilarity); Table S5: Marginal effects of depressive status and covariates on gut microbiota beta diversity (unweighted UniFrac distances); Table S6: Marginal effects of depressive status and covariates on gut microbiota beta diversity (weighted UniFrac distances).
