# Supplementary material for: Integrated Inflammatory and Gut Microbial Signatures in Major Depressive Disorder: A Case–Control Study
Source: Brain Sci. 2026 Jun 28;16(7):681. doi: 10.3390/brainsci16070681 (PMC13407155; doi:10.3390/brainsci16070681)
Supplement: Supplementary file 1 [file brainsci-16-00681-s001.zip › brainsci-4309552-supplementary.pdf]

Supplementary Table S1. Differential abundance of bacterial genera in depressed patients versus healthy controls.

Relative abundance- Genus					
		**All participants (n=71)**	**Depressed (n=46)**	**Healthy controls (n=25)**	**P value**
Prevotella	*Mean ± SD*	-0.860 ± 0.732	-0.831 ± 0.650	-0.915 ± 0.874	0.648
Phocaeicola	*Mean ± SD*	-1.816 ± 1.009	-1.869 ± 1.139	-1.719 ± 0.723	0.555
Faecalibacterium	*Mean ± SD*	-1.428 ± 0.661	-1.490 ± 0.802	-1.315 ± 0.215	0.288
Succinivibrio	*Mean ± SD*	-4.090 ± 2.324	-4.296 ± 2.276	-3.711 ± 2.410	0.314
Alistipes	*Mean ± SD*	-2.395 ± 1.438	-2.409 ± 1.417	-2.370 ± 1.505	0.916
Parabacteroides	*Mean ± SD*	-2.291 ± 0.896	-2.262 ± 1.033	-2.345 ± 0.579	0.714
Bacteroides	*Mean ± SD*	-1.843 ± 0.845	-1.873 ± 0.910	-1.787 ± 0.725	0.685
Eubacterium	*Mean ± SD*	-1.803 ± 0.493	-1.812 ± 0.521	-1.786 ± 0.444	0.830
Clostridium	*Mean ± SD*	-3.523 ± 1.259	-3.672 ± 1.354	-3.248 ± 1.032	0.177
Ruminococcus	*Mean ± SD*	-2.227 ± 0.992	-2.244 ± 1.037	-2.195 ± 0.921	0.845
Alloprevotella	*Mean ± SD*	-3.731 ± 2.081	-3.539 ± 2.067	-4.086 ± 2.102	0.293
Roseburia	*Mean ± SD*	-2.120 ± 0.763	-2.241 ± 0.892	-1.897 ± 0.353	0.069
Bifidobacterium	*Mean ± SD*	-3.065 ± 1.335	-3.216 ± 1.528	-2.785 ± 0.835	0.195
Dialister	*Mean ± SD*	-1.963 ± 1.107	-1.819 ± 1.046	-2.227 ± 1.189	0.139
Prevotellamassilia	*Mean ± SD*	-4.199 ± 2.174	-4.322 ± 2.152	-3.971 ± 2.241	0.519
Gemmiger	*Mean ± SD*	-2.320 ± 0.761	-2.376 ± 0.901	-2.216 ± 0.388	0.404
Butyrivibrio	*Mean ± SD*	-4.833 ± 1.685	-4.889 ± 1.668	-4.730 ± 1.746	0.707
Dorea	*Mean ± SD*	-2.484 ± 0.551	-2.397 ± 0.601	-2.645 ± 0.407	0.070
Megasphaera	*Mean ± SD*	-3.948 ± 1.982	-4.065 ± 2.084	-3.732 ± 1.798	0.503
Romboutsia	*Mean ± SD*	-3.534 ± 1.507	-3.632 ± 1.538	-3.354 ± 1.463	0.461
Dysosmobacter	*Mean ± SD*	-4.143 ± 1.481	-3.949 ± 1.443	-4.499 ± 1.515	0.137
Blautia	*Mean ± SD*	-2.328 ± 0.453	-2.262 ± 0.468	-2.451 ± 0.404	0.093
Selenomonas	*Mean ± SD*	-5.622 ± 1.260	-5.610 ± 1.288	-5.645 ± 1.231	0.914
Coprococcus	*Mean ± SD*	-2.231 ± 0.580	-2.226 ± 0.378	-2.241 ± 0.845	0.916
Marseillibacter	*Mean ± SD*	-2.783 ± 1.116	-2.952 ± 1.235	-2.473 ± 0.785	0.084

Treponema	*Mean ± SD*	-5.686 ± 1.152	-5.796 ± 0.967	-5.482 ± 1.433	0.276
Phascolarctobacterium	*Mean ± SD*	-4.577 ± 1.760	-4.487 ± 1.821	-4.743 ± 1.664	0.562
Elusimicrobium	*Mean ± SD*	-5.186 ± 1.573	-5.432 ± 1.262	-4.734 ± 1.975	0.074
Mediterraneibacter	*Mean ± SD*	-2.929 ± 0.983	-2.747 ± 0.838	-3.264 ± 1.150	**0.033**
Odoribacter	*Mean ± SD*	-3.354 ± 1.345	-3.517 ± 1.405	-3.054 ± 1.197	0.168
[Eubacterium]_eligens_group	*Mean ± SD*	-3.286 ± 1.426	-3.593 ± 1.589	-2.722 ± 0.830	**0.013**
Catenibacterium	*Mean ± SD*	-4.148 ± 1.870	-3.983 ± 1.900	-4.451 ± 1.812	0.318
Barnesiella	*Mean ± SD*	-3.491 ± 1.693	-3.638 ± 1.752	-3.222 ± 1.576	0.327
Akkermansia	*Mean ± SD*	-5.091 ± 1.537	-5.146 ± 1.597	-4.991 ± 1.446	0.689
Porphyromonas	*Mean ± SD*	-5.671 ± 0.839	-5.719 ± 0.741	-5.584 ± 1.007	0.520
Sutterella	*Mean ± SD*	-3.344 ± 1.662	-3.559 ± 1.653	-2.948 ± 1.639	0.140
Fusicatenibacter	*Mean ± SD*	-3.483 ± 1.212	-3.416 ± 1.187	-3.607 ± 1.271	0.530
Mitsuokella	*Mean ± SD*	-4.562 ± 1.706	-4.912 ± 1.647	-3.917 ± 1.652	**0.018**
Oscillibacter	*Mean ± SD*	-3.321 ± 1.430	-3.382 ± 1.494	-3.209 ± 1.326	0.629
Anaerotignum	*Mean ± SD*	-3.265 ± 0.933	-3.340 ± 1.103	-3.127 ± 0.476	0.362
Megamonas	*Mean ± SD*	-4.784 ± 1.770	-4.584 ± 1.905	-5.153 ± 1.455	0.198
Paraprevotella	*Mean ± SD*	-4.283 ± 1.703	-4.293 ± 1.765	-4.263 ± 1.619	0.944
Acidaminococcus	*Mean ± SD*	-5.079 ± 1.571	-5.077 ± 1.613	-5.082 ± 1.523	0.991
Faecalibacillus	*Mean ± SD*	-4.044 ± 1.437	-4.092 ± 1.484	-3.955 ± 1.371	0.704
[Eubacterium]	*Mean ± SD*	-4.398 ± 1.640	-4.128 ± 1.638	-4.896 ± 1.555	0.059
Desulfovibrio	*Mean ± SD*	-3.800 ± 1.581	-3.809 ± 1.702	-3.785 ± 1.361	0.951
Sporobacter	*Mean ± SD*	-3.480 ± 1.522	-3.540 ± 1.602	-3.370 ± 1.386	0.656
Duodenibacillus	*Mean ± SD*	-4.246 ± 1.668	-4.122 ± 1.594	-4.476 ± 1.807	0.397
Parasutterella	*Mean ± SD*	-4.338 ± 1.549	-4.494 ± 1.523	-4.052 ± 1.587	0.254
[Ruminococcus]_torques_group	*Mean ± SD*	-4.760 ± 1.525	-4.682 ± 1.547	-4.905 ± 1.504	0.560
[Eubacterium]_hallii_group	*Mean ± SD*	-3.383 ± 1.151	-3.202 ± 1.084	-3.716 ± 1.216	0.072
Bilophila	*Mean ± SD*	-4.176 ± 1.439	-3.934 ± 1.467	-4.623 ± 1.297	0.053
Massiliprevotella	*Mean ± SD*	-4.710 ± 1.742	-4.812 ± 1.753	-4.521 ± 1.742	0.504
Streptococcus	*Mean ± SD*	-3.854 ± 1.324	-4.066 ± 1.489	-3.466 ± 0.843	0.068
Lachnospiraceae_UCG-004	*Mean ± SD*	-3.512 ± 1.346	-3.558 ± 1.352	-3.427 ± 1.359	0.698

Collinsella	*Mean ± SD*	-4.117 ± 1.417	-3.670 ± 1.253	-4.939 ± 1.353	**0.000**
Ligilactobacillus	*Mean ± SD*	-4.359 ± 1.593	-4.509 ± 1.630	-4.083 ± 1.515	0.284
Lachnoclostridium	*Mean ± SD*	-4.569 ± 1.490	-4.085 ± 1.455	-5.461 ± 1.106	**0.000**
Alteracholeplasma	*Mean ± SD*	-5.074 ± 1.340	-5.323 ± 1.166	-4.615 ± 1.532	**0.032**
Flavonifractor	*Mean ± SD*	-5.166 ± 1.329	-5.085 ± 1.358	-5.316 ± 1.286	0.487
Anaerostipes	*Mean ± SD*	-3.454 ± 1.202	-3.368 ± 1.234	-3.614 ± 1.148	0.415
Agathobaculum	*Mean ± SD*	-3.525 ± 1.285	-3.374 ± 1.168	-3.803 ± 1.459	0.181
Paludicola	*Mean ± SD*	-4.261 ± 1.320	-4.517 ± 1.299	-3.791 ± 1.249	**0.026**
Methanobrevibacter	*Mean ± SD*	-4.813 ± 1.492	-4.674 ± 1.570	-5.070 ± 1.329	0.288
Christensenella	*Mean ± SD*	-3.987 ± 1.548	-4.344 ± 1.467	-3.330 ± 1.504	**0.008**
Ruthenibacterium	*Mean ± SD*	-5.067 ± 1.347	-4.974 ± 1.395	-5.238 ± 1.264	0.434
Flintibacter	*Mean ± SD*	-3.720 ± 1.122	-3.934 ± 1.194	-3.325 ± 0.865	**0.028**
Lactobacillus	*Mean ± SD*	-4.414 ± 1.317	-4.413 ± 1.356	-4.416 ± 1.270	0.993
Acinetobacter	*Mean ± SD*	-5.837 ± 0.608	-5.787 ± 0.707	-5.929 ± 0.355	0.352
Turicibacter	*Mean ± SD*	-4.889 ± 1.338	-4.924 ± 1.328	-4.824 ± 1.382	0.764
Klebsiella	*Mean ± SD*	-5.421 ± 1.257	-5.186 ± 1.420	-5.855 ± 0.727	**0.031**
Intestinibacter	*Mean ± SD*	-5.033 ± 1.341	-4.882 ± 1.370	-5.310 ± 1.267	0.201
Coprobacter	*Mean ± SD*	-4.990 ± 1.397	-5.138 ± 1.292	-4.717 ± 1.565	0.227
Lawsonibacter	*Mean ± SD*	-5.275 ± 1.184	-5.080 ± 1.294	-5.634 ± 0.859	0.059
Duncaniella	*Mean ± SD*	-5.697 ± 1.010	-5.844 ± 0.741	-5.427 ± 1.353	0.097
Citrobacter	*Mean ± SD*	-5.769 ± 0.865	-5.800 ± 0.773	-5.712 ± 1.028	0.686
Neglectibacter	*Mean ± SD*	-4.690 ± 1.295	-4.604 ± 1.319	-4.847 ± 1.261	0.456
Tyzzerella	*Mean ± SD*	-5.288 ± 1.269	-5.172 ± 1.374	-5.503 ± 1.042	0.296
Allisonella	*Mean ± SD*	-4.750 ± 1.435	-4.576 ± 1.480	-5.070 ± 1.316	0.167
Slackia	*Mean ± SD*	-5.299 ± 1.237	-5.140 ± 1.343	-5.591 ± 0.972	0.143
Cutibacterium	*Mean ± SD*	-5.777 ± 0.692	-5.857 ± 0.556	-5.629 ± 0.885	0.187
Fusobacterium	*Mean ± SD*	-5.615 ± 1.098	-5.712 ± 0.957	-5.438 ± 1.322	0.320
Victivallis	*Mean ± SD*	-5.181 ± 1.325	-5.538 ± 1.036	-4.525 ± 1.553	**0.002**
Oxalobacter	*Mean ± SD*	-4.285 ± 1.394	-4.335 ± 1.387	-4.193 ± 1.430	0.684
Veillonella	*Mean ± SD*	-4.740 ± 1.399	-4.690 ± 1.426	-4.830 ± 1.371	0.690

Howardella	*Mean ± SD*	-5.137 ± 1.191	-4.924 ± 1.254	-5.529 ± 0.970	**0.040**
Paramuribaculum	*Mean ± SD*	-5.835 ± 0.708	-5.906 ± 0.637	-5.705 ± 0.820	0.255
Butyricimonas	*Mean ± SD*	-3.463 ± 1.307	-3.653 ± 1.332	-3.112 ± 1.206	0.096
Terrisporobacter	*Mean ± SD*	-5.363 ± 1.198	-5.379 ± 1.198	-5.333 ± 1.223	0.879
Weissella	*Mean ± SD*	-5.812 ± 0.628	-5.799 ± 0.665	-5.837 ± 0.566	0.813
Olsenella	*Mean ± SD*	-5.408 ± 1.182	-5.498 ± 1.137	-5.242 ± 1.268	0.389
Colidextribacter	*Mean ± SD*	-4.716 ± 1.365	-4.744 ± 1.399	-4.663 ± 1.327	0.813
Hungatella	*Mean ± SD*	-5.656 ± 0.984	-5.663 ± 0.991	-5.643 ± 0.991	0.934
Haemophilus	*Mean ± SD*	-5.151 ± 1.296	-5.195 ± 1.252	-5.072 ± 1.397	0.705
Parafannyhessea	*Mean ± SD*	-5.720 ± 0.858	-5.750 ± 0.824	-5.664 ± 0.932	0.692
Intestinimonas	*Mean ± SD*	-4.556 ± 1.343	-4.700 ± 1.398	-4.291 ± 1.219	0.224
Limosilactobacillus	*Mean ± SD*	-5.779 ± 0.815	-5.724 ± 0.914	-5.881 ± 0.593	0.440
Enterococcus	*Mean ± SD*	-5.739 ± 0.813	-5.815 ± 0.733	-5.598 ± 0.944	0.286
Senegalimassilia	*Mean ± SD*	-4.819 ± 1.343	-4.685 ± 1.413	-5.066 ± 1.192	0.256
Pseudomonas	*Mean ± SD*	-5.409 ± 1.075	-5.522 ± 0.996	-5.201 ± 1.200	0.231
Monoglobus	*Mean ± SD*	-5.217 ± 1.244	-5.133 ± 1.292	-5.372 ± 1.158	0.443
Staphylococcus	*Mean ± SD*	-5.804 ± 0.653	-5.796 ± 0.670	-5.819 ± 0.634	0.889
Eisenbergiella	*Mean ± SD*	-5.810 ± 0.714	-5.806 ± 0.760	-5.818 ± 0.637	0.946
Oribacterium	*Mean ± SD*	-5.775 ± 0.834	-5.702 ± 0.978	-5.909 ± 0.453	0.319
Petroclostridium	*Mean ± SD*	-5.602 ± 1.007	-5.637 ± 0.964	-5.539 ± 1.098	0.699
Caproicibacter	*Mean ± SD*	-5.404 ± 1.060	-5.484 ± 1.015	-5.256 ± 1.145	0.390
Comamonas	*Mean ± SD*	-5.745 ± 0.731	-5.738 ± 0.766	-5.757 ± 0.678	0.916
Anaerotruncus	*Mean ± SD*	-5.338 ± 1.121	-5.251 ± 1.165	-5.498 ± 1.039	0.380
Campylobacter	*Mean ± SD*	-5.669 ± 0.857	-5.816 ± 0.613	-5.399 ± 1.149	**0.049**
RF39	*Mean ± SD*	-4.790 ± 1.429	-4.772 ± 1.454	-4.822 ± 1.409	0.889
Adlercreutzia	*Mean ± SD*	-5.438 ± 1.065	-5.410 ± 1.083	-5.490 ± 1.050	0.766
Bulleidia	*Mean ± SD*	-5.455 ± 1.077	-5.608 ± 0.956	-5.173 ± 1.243	0.105
Ihubacter	*Mean ± SD*	-5.072 ± 1.203	-4.970 ± 1.254	-5.260 ± 1.104	0.335
Butyricicoccus	*Mean ± SD*	-5.579 ± 0.948	-5.512 ± 1.008	-5.702 ± 0.832	0.424
Negativibacillus	*Mean ± SD*	-5.675 ± 0.877	-5.498 ± 1.052	-6.000 ± 0.000	**0.020**

Coprobacillus	*Mean ± SD*	-5.735 ± 0.830	-5.817 ± 0.717	-5.583 ± 1.004	0.261
Raoultibacter	*Mean ± SD*	-5.603 ± 0.902	-5.492 ± 0.987	-5.807 ± 0.690	0.162
Eggerthella	*Mean ± SD*	-5.723 ± 0.804	-5.658 ± 0.912	-5.841 ± 0.550	0.363
Erysipelatoclostridium	*Mean ± SD*	-5.839 ± 0.669	-5.751 ± 0.821	-6.000 ± 0.000	0.135
Cerasicoccus	*Mean ± SD*	-5.786 ± 0.713	-5.833 ± 0.643	-5.701 ± 0.833	0.461
Anaeromassilibacillus	*Mean ± SD*	-5.525 ± 0.937	-5.398 ± 1.038	-5.759 ± 0.670	0.122
Aminipila	*Mean ± SD*	-5.644 ± 0.846	-5.565 ± 0.897	-5.787 ± 0.737	0.294
Mailhella	*Mean ± SD*	-5.825 ± 0.649	-5.848 ± 0.581	-5.783 ± 0.770	0.689
Tractidigestivibacter	*Mean ± SD*	-5.830 ± 0.630	-5.835 ± 0.640	-5.820 ± 0.625	0.920
Cloacibacillus	*Mean ± SD*	-5.866 ± 0.553	-5.844 ± 0.597	-5.906 ± 0.472	0.659
Hydrogeniiclostridium	*Mean ± SD*	-5.841 ± 0.589	-5.754 ± 0.720	-6.000 ± 0.000	0.093
Fenollaria	*Mean ± SD*	-5.741 ± 0.745	-5.762 ± 0.691	-5.703 ± 0.849	0.753
Faecalicatena	*Mean ± SD*	-5.907 ± 0.452	-5.895 ± 0.499	-5.929 ± 0.356	0.768
Parolsenella	*Mean ± SD*	-5.761 ± 0.736	-5.718 ± 0.821	-5.840 ± 0.554	0.506
Lactococcus	*Mean ± SD*	-5.796 ± 0.687	-5.867 ± 0.512	-5.667 ± 0.927	0.245
Gabonibacter	*Mean ± SD*	-5.799 ± 0.672	-5.786 ± 0.706	-5.823 ± 0.620	0.828
Phocea	*Mean ± SD*	-5.858 ± 0.526	-5.819 ± 0.599	-5.929 ± 0.353	0.401
Holdemania	*Mean ± SD*	-5.805 ± 0.652	-5.744 ± 0.748	-5.918 ± 0.412	0.288
Hydrogenoanaerobacterium	*Mean ± SD*	-5.885 ± 0.476	-5.957 ± 0.293	-5.752 ± 0.686	0.083
Parvimonas	*Mean ± SD*	-5.816 ± 0.615	-5.809 ± 0.631	-5.829 ± 0.596	0.897
Corynebacterium	*Mean ± SD*	-5.875 ± 0.523	-5.911 ± 0.421	-5.807 ± 0.676	0.427
Beduinibacterium	*Mean ± SD*	-5.869 ± 0.484	-5.872 ± 0.495	-5.864 ± 0.471	0.949
Gemella	*Mean ± SD*	-5.831 ± 0.570	-5.739 ± 0.694	-6.000 ± 0.000	0.065
Harryflintia	*Mean ± SD*	-5.883 ± 0.484	-5.819 ± 0.594	-6.000 ± 0.000	0.134

P-values in bold indicate statistically significant differences between depressed participants and healthy controls, based on a threshold of $p < 0.05$

Supplementary Table S2. Differential abundance of bacterial phyla in depressed patients versus healthy controls.

Relative abundance- Phylum					
		**All participants (n=71)**	**Depressed (n=46)**	**Healthy controls (n=25)**	**P value**
Actinobacteria	*Mean ± SD*	-2.433 ± 0.826	-2.423 ± 0.965	-2.452 ± 0.496	0.892
Bacteroidetes	*Mean ± SD*	-0.366 ± 0.193	-0.373 ± 0.225	-0.352 ± 0.114	0.672
Candidatus_Thermoplasmatota	*Mean ± SD*	-5.967 ± 0.274	-6.000 ± 0.000	-5.908 ± 0.462	0.177
Cyanobacteria	*Mean ± SD*	-5.302 ± 1.215	-5.013 ± 1.370	-5.835 ± 0.572	**0.006**
Deinococcus-Thermus	*Mean ± SD*	-5.967 ± 0.281	-5.948 ± 0.350	-6.000 ± 0.000	0.465
Elusimicrobia	*Mean ± SD*	-5.186 ± 1.573	-5.432 ± 1.262	-4.734 ± 1.975	0.074
Euryarchaeota	*Mean ± SD*	-4.813 ± 1.493	-4.673 ± 1.571	-5.070 ± 1.329	0.288
Firmicutes	*Mean ± SD*	-0.399 ± 0.174	-0.380 ± 0.168	-0.434 ± 0.184	0.213
Fusobacteria	*Mean ± SD*	-5.577 ± 1.132	-5.712 ± 0.957	-5.328 ± 1.387	0.175
Lentisphaerae	*Mean ± SD*	-5.181 ± 1.325	-5.538 ± 1.036	-4.525 ± 1.553	**0.002**
Proteobacteria	*Mean ± SD*	-1.435 ± 0.491	-1.509 ± 0.482	-1.298 ± 0.487	0.084
Spirochaetes	*Mean ± SD*	-5.650 ± 1.182	-5.741 ± 1.026	-5.482 ± 1.433	0.383
Synergistetes	*Mean ± SD*	-5.712 ± 0.767	-5.738 ± 0.761	-5.663 ± 0.791	0.699
Tenericutes	*Mean ± SD*	-4.677 ± 1.436	-5.026 ± 1.307	-4.035 ± 1.466	**0.005**
Verrucomicrobia	*Mean ± SD*	-4.726 ± 1.629	-4.804 ± 1.668	-4.583 ± 1.580	0.590

P-values in bold indicate statistically significant differences between depressed participants and healthy controls, based on a threshold of $p < 0.05$

Supplementary Table S3. Marginal effects of depressive status and covariates on gut microbiota beta diversity (Bray–Curtis dissimilarity).

Variable	R^2	p-value
**Status**	0.021	0.067
**Age**	0.019	0.101
**Sex**	0.021	0.059
**BMI**	0.013	0.704
**Meddiet**	0.016	0.343
**Fluoxetine doses**	0.014	0.587

Supplementary Table S4. Marginal effects of depressive status and covariates on gut microbiota beta diversity (Jaccard dissimilarity).

Variable	R^2	p-value
**Status**	0.057	0.001
**Age**	0.017	0.093
**Sex**	0.013	0.381
**BMI**	0.013	0.425
**Meddiet**	0.014	0.276
**Fluoxetine doses**	0.014	0.276

Supplementary Table S5. Marginal effects of depressive status and covariates on gut microbiota beta diversity (unweighted UniFrac distances).

Variable	R^2	p-value
**Status**	0.02074	0.052
**Age**	0.0192	0.104
**Sex**	0.02159	0.048
**BMI**	0.01279	0.725
**Meddiet**	0.01575	0.339
**Fluoxetine doses**	0.01368	0.592

Supplementary Table S6. Marginal effects of depressive status and covariates on gut microbiota beta diversity (weighted UniFrac distances).

Variable	Sum of Squares	R^2	p-value
**Status**	0.0736	0.02	0.211
**Age**	0.0846	0.023	0.168
**Sex**	0.0538	0.015	0.391
**BMI**	0.078	0.021	0.185
**Meddiet**	0.0185	0.005	0.969
**Fluoxetine doses**	0.0764	0.021	0.198

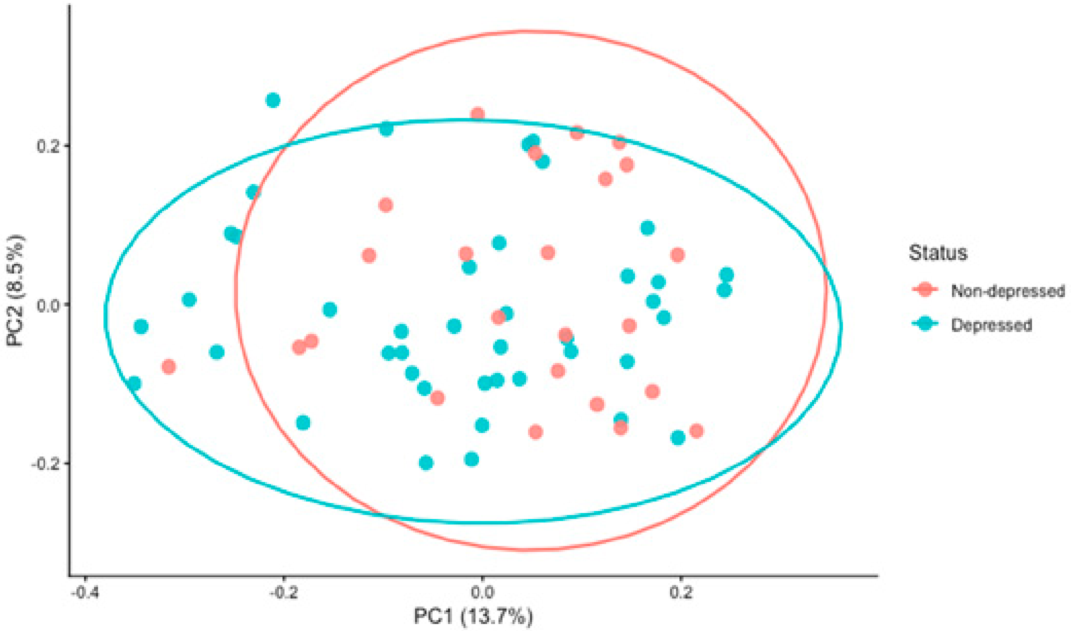

Supplementary Figure S1. Principal Coordinates Analysis (PCoA) of gut microbiota composition (Bray–Curtis distance) Statistical differences were assessed using marginal PERMANOVA adjusted for confounders.

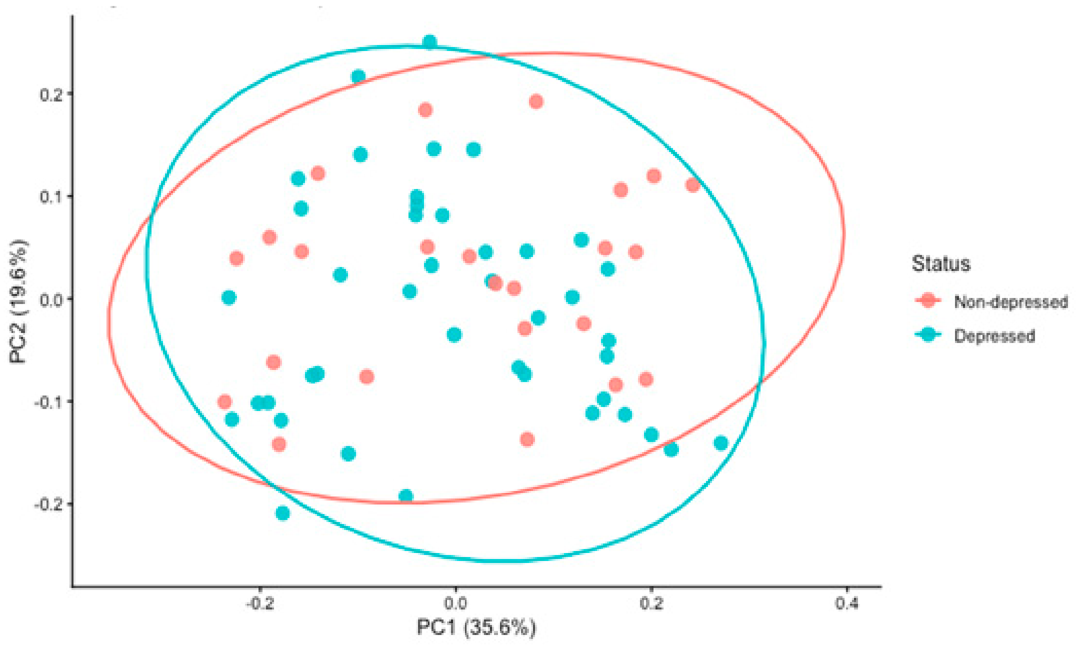

Supplementary Figure S2. Principal Coordinates Analysis (PCoA) of gut microbiota composition (Weighted UniFrac distance) Statistical differences were assessed using marginal PERMANOVA adjusted for confounders.

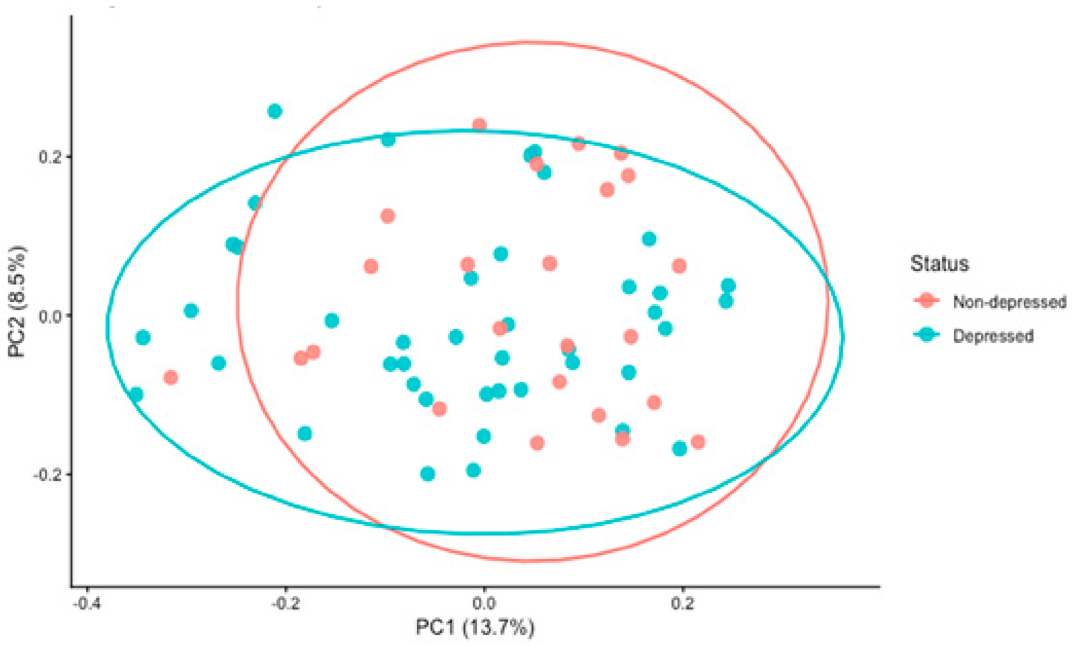

Supplementary Figure S3. Principal Coordinates Analysis (PCoA) of gut microbiota composition (Unweighted UniFrac distance). Statistical differences were assessed using marginal PERMANOVA adjusted for confounders.
